# Domestication and genome evolution in *Allium* crops: From hybrid origins to breeding perspectives

**DOI:** 10.1016/j.isci.2026.114922

**Published:** 2026-02-06

**Authors:** Esther A. Harding, Anahita Karbasi, Alisdair R. Fernie, Mustafa Bulut

**Affiliations:** 1 Program Center MetaCom, Leibniz Institute of Plant Biochemistry, Weinberg 3, 06120 Halle (Saale), Germany; 2 Max-Planck Institute of Molecular Plant Physiology, Am Mühlenberg 1, 14476 Potsdam, Germany

**Keywords:** Plant biology, Plant Genetics, Plant evolution

## Abstract

The *Allium* genus encompasses a range of globally significant vegetables, each shaped by distinct domestication pathways despite shared evolutionary ancestry. Among the most prominent are onion (*Allium cepa*), Welsh onion (*A. fistulosum*), and garlic (*A. sativum*), which exhibit striking differences in morphology, reproductive strategy, and flavor chemistry. Recent advances in high-quality genome assemblies have illuminated the molecular and structural changes underlying their evolution. This review integrates current findings on genome architecture, domestication traits, and key developmental pathways to elucidate how natural and artificial selection have directed their divergent phenotypes. These insights not only clarify the evolutionary history of *Allium* crops but also provide valuable tools for breeding programs aimed at improving resilience, yield, and flavor.

## Graphical abstract

**Figure:**
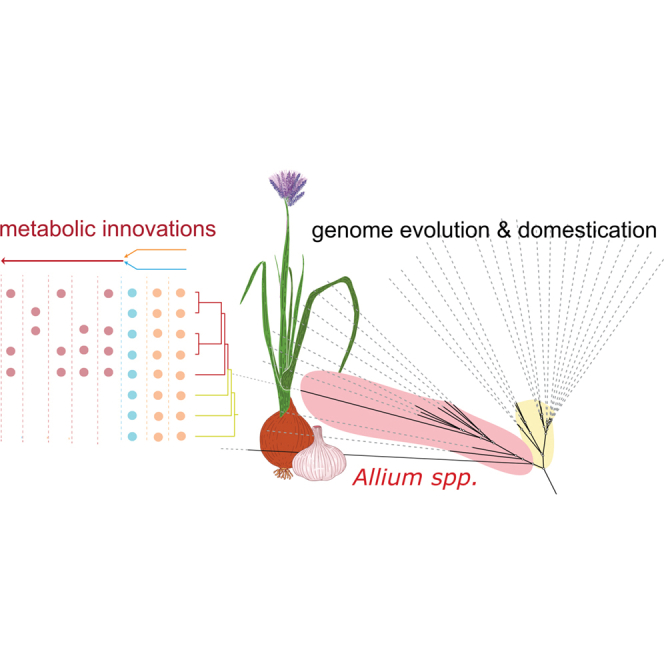

## Introduction

Crop domestication, a pivotal process in agricultural evolution, began about 12,000 years ago and transformed a limited number of wild species into the major crops sustaining today’s food systems.^1^^,^^2^ This transition, from early cultivation and unconscious trait selection to deliberate breeding, was accompanied by genetic bottlenecks and reduced allelic diversity.^3^^,^^4^ While traditionally described through morphological changes such as loss of shattering, increased fruit size, and reduced dormancy,^5^^,^^6^ recent omics studies have revealed species-specific reprogramming of plant metabolism as both a direct and collateral outcome of selection.^3^^,^^7^ Despite the economic relevance of *Allium* crops, their domestication remains less comprehensively studied compared with other species.

The natural distribution of *Allium* spans the Holarctic, with its main diversity center between the Mediterranean, Central Asia, and western China, and a secondary one in North America. Some species extend into the tropical highlands of Asia and Africa, while isolated South American reports are likely misattributions. *Allium synnotii* from South Africa is considered a human introduction followed by hybridization and polyploidization.^8^^,^^9^^,^^10^ *Allium* genomes, among the larger ones in flowering plants, are inflated by transposable element activity and slow DNA elimination but retain conserved gene content and macrosynteny, facilitating comparative studies.^11^ Domestication likely progressed from wild plant use to cultivation, producing the morphological and genetic diversity of modern *Allium* crops. While the historical cultivation of onion and garlic is well documented, questions remain regarding their precise phylogenetic origins and whether domestication was singular or occurred multiple times in different regions.

**Figure 1:**
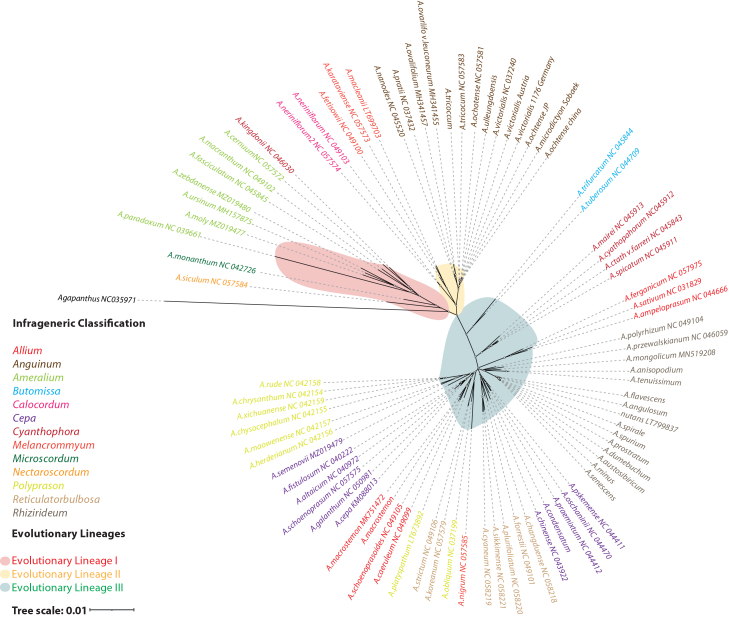
Phylogenetic tree of the major evolutionary lineages in the *Allium* genus Maximum likelihood analysis of 76 *Allium* species covering the three main evolutionary lineages. The color of the phylogeny reflects the evolutionary lineages, respectively, while the species color reflects the intergeneric classification. The chloroplast sequences for the phylogeny were retrieved from Yang et al.^32^

Moreover, molecular phylogenies have reshaped *Allium* classification into three major lineages encompassing 15 subgenera and 72 sections^9^ (Figure 1). The earliest lineage includes Nectaroscordum, Microscordum, and Amerallium, characterized by bulbous or rhizomatous growth and basic chromosome numbers of x = 7–9. The second lineage, including Anguinum, is typically rhizomatous with x = 8.^12^ Despite progress, taxonomic challenges persist, with paraphyletic groups and evidence of hybridization and polyploidy causing discordance between nuclear and plastid data.^13^^,^^14^^,^^15^^,^^16^^,^^17^^,^^18^^,^^19^^,^^20^ Resolving these discrepancies requires more integrative analyses that combine nuclear and plastid genomic frameworks.^21^^,^^22^ These phylogenetic uncertainties are compounded by ongoing debates surrounding the evolutionary timeline of the genus, ranging from 11 to 52.2 million years, depending on molecular dating methods and the lack of fossil evidence.^15^^,^^16^^,^^18^^,^^23^^,^^24^^,^^25^^,^^26^ The most supported estimate places divergence at ∼52.2 million years ago.^24^^,^^27^ Biogeographic reconstructions suggest an Indian subcontinent origin, followed by dispersal throughout Asia and repeated entries into North America via the Bering Land Bridge, exemplified by *A. schoenoprasum*.^24^^,^^28^^,^^29^^,^^30^^,^^31^

Domestication-related relationships have further been illuminated by plastome-based phylogenies of four Central Asian species (*A. oschaninii*, *A. praemixtum*, *A. pskemense*, and *A. galanthum*), which form two principal clades. One comprises the first three species, while the second includes *A. galanthum*, *A. altaicum*, and the cultivated *A. cepa* and *A. fistulosum.*^21^ This suggests an independent domestication of Central Asian onion species, contradicting earlier ITS and morphological studies, possibly due to chloroplast capture.^20^^,^^33^^,^^34^^,^^35^ Subsequent analysis indicated *A. fistulosum* as monophyletic from *A. altaicum*, with domestication-associated bottlenecks reducing its genetic diversity.^36^ In *A. sativum*, infraspecific differentiation revealed four major groups, including *A. longicuspis*, which harbors numerous intermediate forms thought to be progenitors of cultivated lineages.^37^

Although the nuclear genomes sequencing remains difficult for most *Allium* species, due to their large genome size, substantial advances have been made for *A. cepa*, *A. sativum*, and *A. fistulosum*.^11^^,^^38^^,^^39^^,^^40^ Complementary resources, including over 60 plastid genomes and expanding repertoire of multiple transcriptomes, are now enabling finer dissection of hybridogenic evolution, taxonomic boundaries, and domestication trajectories.^17^^,^^18^^,^^19^^,^^41^^,^^42^^,^^43^

## Origins, hybridization, and genome evolution in *Allium* crops

Within the Cepa section, *A. cepa* and its wild relatives (*A. vavilovii*, *A. oschaninii*, and *A. galanthum*) occur mainly in the Irano-Turanian region, while *A. galanthum* extends to higher latitudes, including regions such as Kazakhstan and Altay.^20^^,^^33^^,^^44^ Although molecular studies identify *A. vavilovii* as the closest relative of *A. cepa*, morphological differences suggest a hybrid origin involving multiple progenitors.^20^^,^^34^^,^^45^ The true ancestor remains unresolved, likely obscured by hybridization events. The *A. cepa* genome (∼16.2 Gb) is over 95% repetitive, dominated by Gypsy- and Copia-type LTR retrotransposons, which expanded in bursts and now embed gene-rich regions within heterochromatin.^11^^,^^46^^,^^47^^,^^48^ Despite this, onion retains ∼61,619–65,730 gene models, with expansions in families linked to abiotic stress, carbohydrate metabolism, and flavor traits.^11^^,^^49^

Contrarily, cultivated *A. sativum* and *A. longicuspis*, as its closest wild ancestor, are mostly sterile and morphologically indistinguishable, often treated as synonyms, and originated in Central Asia (Figure 3B). Long-term clonal propagation and regional selection generated three major cultivated groups – Longicuspis, Sativum, and Ophioscorodon – each adapted to specific environments and culinary uses.^50^ The Longicuspis group retains some fertility and genetic diversity, Sativum (softneck and hardneck) dominates temperate regions, and Ophioscorodon is linked to European landraces.^50^ Vegetative propagation likely arose from human selection for traits such as bulb size and earliness.^51^^,^^52^ Fertility can be restored experimentally, but the status of *A. longicuspis* as progenitor versus feral form remains debated,^44^^,^^53^^,^^54^ while *A. tuncelianum* is now considered distantly related.^55^ The garlic genome (∼16.24 Gb) is >90% LTR retrotransposons, with segmental duplications and ancient polyploid-like signatures.^11^^,^^40^ Despite diploidy, its genome shows structural complexity and unique expansions of sulfur metabolism genes (alliinase, γ-glutamyl transpeptidase, cysteine synthase), central to flavor, pest resistance, and medicinal properties.^11^^,^^40^^,^^49^

*A. fistulosum*, widely cultivated in East Asia, derives monophyletically from *A. altaicum*.^36^ In contrast, the Egyptian onion (*A. × proliferum*) is a sterile hybrid between *A. cepa* and *A. fistulosum*, with repeated origins in areas where both parents co-occur.^56^^,^^57^^,^^58^^,^^59^ Its genome (∼11.27 Gb) is likewise TE-rich but collinear with onion, indicating conserved structure.^11^^,^^39^^,^^46^ Lineage-specific TE activity and divergent regulation of cytokinin and auxin signaling genes underpin *A. fistulosum*’s perennial growth and strong tillering,^11^^,^^39^^,^^60^ while leek and allies belong to the *A. ampeloprasum* complex, a polyploid group spanning the Mediterranean and Asia.^57^^,^^61^^,^^62^ Both leek and great-headed garlic are allopolyploids with distinct parental contributions,^10^ though genomic relationships within the complex remain unresolved. The lectotype of *A. ampeloprasum* applies to great-headed garlic, while leek should be classified as *A. porrum*.^27^

Moreover, the Chinese chive (*A. tuberosum*), domesticated in northern China >3,000 years ago, is the second-most important *Allium* in East Asia.^57^ It is distinct from its sister *A. ramosum* despite morphological similarities, with evidence for multiple domestication events or introgression from wild relatives.^63^ Further, looking at the origins, the French gray shallot shows a monophyletic origin, mainly from *A. oschaninii* with contributions from *A. cepa* or *A. vavilovii*^56^ and the sterile triploid *A. × cornutum*, cultivated across Europe, Tibet, and Canada, likely originated monophyletically in the Jammu-Afghanistan-Pakistan region through hybridization among *A. cepa*, *A. roylei*, and possibly *A. pskemense*.^56^^,^^58^^,^^64^

Aside from the taxonomic analysis, recent comparative genomics shows that variation in *Allium* genome size reflects not only TE bursts but also differential LTR retention, silencing, and segmental duplications. High-quality assemblies reveal persistent intact LTRs and locus-specific retention near genes, with methylation and small RNA pathways determining insertion silencing and shaping nearby gene expression.^11^^,^^39^^,^^65^ TE-rich regions correlate with dense CHG/CHH methylation and 24-nt siRNAs, generating epialleles that mimic *cis*-regulatory evolution.^11^ To this end, long-read sequencing-based assembly with Hi-C-assisted scaffolding can be used to resolve allopolyploid origins and chromosome restructuring, exposing subgenome architecture, homeologous exchanges, and duplication events in taxa such as *A. ampeloprasum* and *A. × cornutum*. As recently demonstrated for *A. cepa*, *A. sativum,* and *A. fistulosum*, these analyses aid in resolving the map of sulfur-metabolism gene expansions, revealing duplication modes (tandem, segmental, TE-mediated) critical for distinguishing dosage effects from neofunctionalization. In garlic, duplicated sulfur-related genes show dosage-sensitive expression, with subsets specialized to tissues or stages, underpinning clove-specific metabolite profiles, while others remain broadly expressed, maintaining redundancy and boosting flux.^11^^,^^40^^,^^49^ Furthermore, from a population genomic view, genome-wide association studies (GWASs) and integrated GWAS-transcriptome analyses^39^^,^^65^ linked bulb formation, storability, and photoperiod response to TE-rich haplotypes and expanded gene families, supporting a model where structural variation, gene dosage, and epigenetic regulation collectively shape agronomic traits.

Encompassing polyploidy is a defining feature of *Allium* genome evolution and has contributed substantially to the structural and functional diversification observed across the genus. Garlic and other *Allium* species exhibit extensive genome duplication, including both ancient whole-genome duplication events and lineage-specific segmental polyploidization, which have generated large gene families involved in flowering regulation, stress responses, and organ development.^11^^,^^40^^,^^65^ These duplications, together with massive TE-driven genome expansion, have produced one of the largest diploid genomes among cultivated plants and created abundant substrate for regulatory innovation during domestication. Several key developmental regulators, including PEBP (FT/TFL) and AP2-like genes, show evidence of duplication and neofunctionalization, linking polyploidy-driven gene diversification to shifts in bulb architecture and reproductive capacity.^40^^,^^66^ As domestication favored vegetative propagation, duplicated flowering genes experienced relaxed selection or regulatory divergence, contributing to the polygenic erosion of fertility characteristic of modern cultivars.

## Domestication of *Allium* crops: evolutionary trade-offs and genomic insights

**Figure 2:**
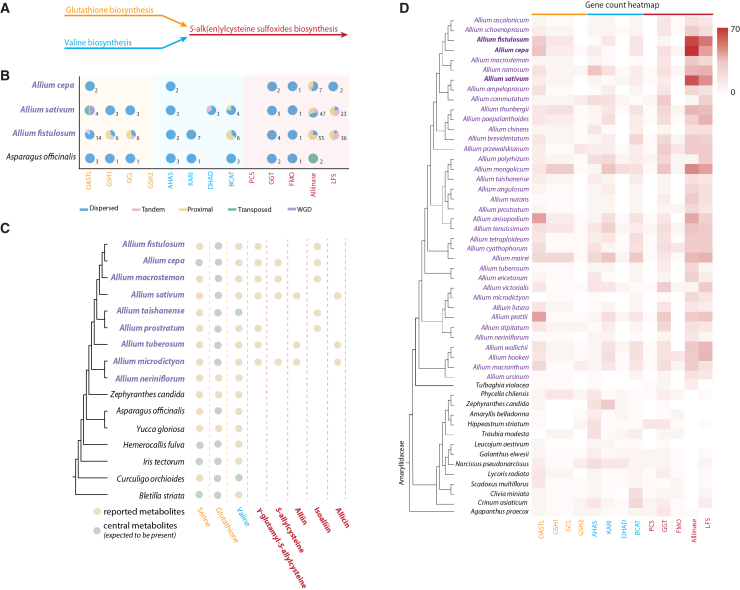
Evolution of the flavor-related genes and metabolites within the *Allium* genus (A) Schematic illustration of the biosynthetic modules for *S*-alk(en)ylcysteine sulfoxides deriving from valine and glutathione biosynthesis. (B) The type of gene duplications involved in the biosynthesis of the flavor-related genes. (C) Reported flavor-related metabolites within 16 Asparagales species based on Wang et al.^67^ (D) Number of gene copies involved in the *S*-alk(en)ylcysteine sulfoxide biosynthesis across several species within the Amaryllidaceae. The color code reflects the affiliation to either biosynthetic module – valine, glutathione, and *S*-alk(en)ylcysteine sulfoxides, respectively. Parts of the figure are modified from to Wang et al.^67^ OASTL = *O*-acetylserine (thiol) lyase; GSH1 = γ-glutamylcysteine synthetase; GCL = γ-glutamylcysteine ligase; GSH2 = glutathione synthetase; AHAS = acetohydroxyacid synthase; KARI = ketol-acid reductoisomerase; DHAD = dihydroxy-acid dehydratase; BCAT = branched-chain amino acid aminotransferase; PCS = phytochelatin synthase; GGT = γ-glutamyl transpeptidases; FMO = flavin-containing monooxygenase; LFS = lachrymatory factor synthase.

Domestication in *Allium* species reflects adaptation to distinct ecological niches, reproductive strategies, and culinary roles. In onion and garlic, selection targeted bulb formation and flavor, while in Welsh onion, emphasis was placed on vegetative vigor and cold tolerance. The evolution of *Allium* flavor traits exemplifies lineage-specific innovation, namely sulfur-metabolism genes underwent expansion and neofunctionalization within the genus, particularly those involved in the biosynthesis of *S*-alk(en)ylcysteine sulfoxides (CSOs), precursors of the distinctive volatile sulfur compounds in garlic and onion (Figures 2A and 2B). Phylotranscriptomic analyses across 501 taxa indicate that while core sulfur pathway genes (OASTL, GSH1, and GCL) are conserved across Asparagales, major expansions of alliinase, γ-glutamyl transpeptidase (GGT), and lachrymatory factor synthase (LFS) occurred specifically in *Allium*.^67^ These expansions, coinciding with the exclusive presence of CSOs in *Allium* (Figures 2B and 2D), traced back to the last common ancestor of the genus and were refined by tandem, dispersed, and proximal duplications (Figure 2C). Adaptive amino acid substitutions in alliinase and LFS suggest functional divergence, possibly linked to enhanced herbivore defense following post-Miocene increases in insect pressure (∼5 Ma). In the following sections, we will focus on the domestication and population genomic insights of the three main *Allium* crops, *A. cepa*, *A. fistulosum,* and *A. sativum*.

*A. sativum* exemplifies domestication-driven reproductive shifts, namely, most cultivars being sterile due to long-term vegetative propagation and selection for bulb traits.^40^^,^^68^^,^^69^ Nonetheless, genomic and transcriptomic studies reveal that garlic retains nearly all flowering gene families. A chromosome-scale assembly published in 2020 identified homologs across photoperiod, vernalization, circadian, meristem identity, floral organ, and pollen-development pathways.^66^^,^^69^ Notably, garlic encodes ∼26 PEBP (phosphatidylethanolamine binding protein) genes, with expanded FT- and TFL1-like clades but lacking a canonical MFT-like clade.^66^^,^^69^ Furthermore, expression studies showed that FT-like homologs vary between vegetative and reproductive tissues, while TFL-like genes are expressed in roots and bulb scales but largely downregulated in reproductive organs. LFY-like genes also show altered regulation, with one homolog (*AsLFY1*) undergoing alternative splicing that correlates with flowering competence.^66^^,^^69^ Overall, these indicate that sterility in mostly cultivated garlic arises from regulatory rewiring rather than complete gene loss.

Moreover, on a population level, resequencing has further clarified garlic domestication. Li et al.^65^ analyzed 230 accessions, identifying ∼129 million variants and distinguishing three major groups: an origin group (OG) and two independently domesticated Chinese groups (CG1, CG2). Both cultivated groups show strong genetic differentiation from OG and reduced nucleotide diversity, reflecting bottlenecks and drift. Further, transcriptome analyses revealed that ∼15–18% of genes are differentially expressed between OG and cultivated groups, particularly those linked to bulb traits. Many differentially expressed genes identified lie outside sweep regions, suggesting that changes in regulation, namely via *cis*-elements, splicing, or epigenetics, play a major role in domestication. Candidate genes include an ELF4-like regulator of bulb weight, FT-like genes such as *Asa6G06199.1*, and gibberellin pathway components,^65^ suggesting that flowering-related regulators were co-opted to modulate bulb growth. In addition, population genetics also revealed partial purging of deleterious mutations in selective sweep regions, showing that selection operates effectively even under clonal propagation.

**Figure 3:**
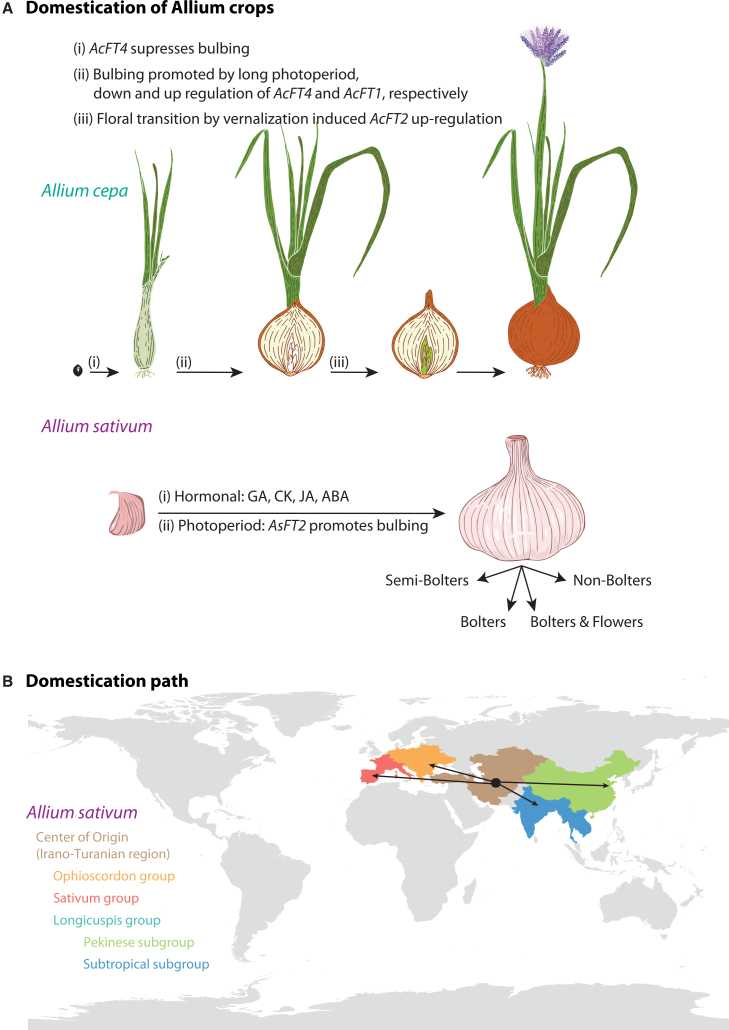
Domestication of Allium crops and their path (A) Overview of domestication-mediated influence of photoperiod in bulbing and flowering in *Allium cepa* and bulbing in *A. sativum*, respectively. The *A. cepa* illustration is modified according to Bulut.^49^ (B) Schematic illustration of the *Allium sativum* domestication path, starting with the dispersal from the center of origin, the Irano-Turanian region, roughly 10,000 years ago, and its introduction to the Mediterranean region, India, and China around 3,000 years ago. FT = *FLOWERING LOCUS T*; GA = gibberellins; CK = cytokinins; JA = jasmonates; ABA = abscisic acid.

In *A. cepa*, domestication reshaped the delicate balance between bulbing and flowering. FT-like genes play a central role, such as *AcFT2,* promoting floral transition, while *AcFT1 and AcFT4* promote bulbing^70^^,^^71^ (Figure 3A). Selection has favored the suppression of *AcFT2* to delay bolting and upregulation of *AcFT1* to enhance bulb yield (Table 1). Comparative genomics shows domestication-related haplotype diversity in *AcFT1/AcFT4* regulatory regions and their downstream MADS-box targets.^71^ This antagonistic regulation highlights the pleiotropy of FT-like genes, delaying flowering to improve bulb size but constraining reproduction, creating a domestication bottleneck. Modern cultivars, therefore, maximize vegetative biomass but often suffer reduced seed set, complicating breeding. FT regulation is also modulated by temperature and hormones (gibberellins and cytokinins), integrating environmental signals into photoperiodic responses, while domestication has favored photoperiod-independent bulbing, enabling wider geographic cultivation. Moreover, genomic diversity studies underscore the population structure of the domesticated onion. To this end, Taylor et al.^72^ showed that photoperiod adaptation strongly structures variation into long-day, intermediate-day, and short-day groups, which act as barriers to gene flow. Further, they identified novel alleles for Fusarium basal rot resistance and seedling vigor, with marker-trait associations found within *A. cepa*, reducing reliance on introgression from wild relatives. Complementary SSR-based analyses of accessions across different countries^73^ confirmed that onion’s obligate outcrossing generates high within-population variation but strong geographical structuring, with Indian accessions forming distinct clusters. From another point, the geographical structuring was reinforced by the recent SNP-based global analysis of multiplier-onion accessions by Jia et al.,^74^ which detected strong eco-geographical clustering, clear stratification by daylength class, and parallel domestication signals across China, including selection signatures in pathways related to bulb enlargement, hormonal regulation, and flowering-time control. Their study also revealed reduced effective population sizes in elite breeding material and evidence of drift and allele erosion relative to landrace diversity, indicating a progressive narrowing of genetic bases in modern cultivars.

**Table 1:** Comparative overview of the divergence and convergence in the domestication of *Allium* crops

Species	Propagation mode	Key domestication traits	Flowering regulatory changes	Sulfur-metabolism duplications	TE/epigenetic role	Breeding implications
*A. cepa*	Sexual (seed-based)	Bulb enlargement, delayed bolting, and photoperiod flexibility	*Cis*-regulatory attenuation of AcFT1; selection for AcFT2 expression (uncoupling bulbing vs. flowering)	Moderate expansions; lineage-specific paralogs	TE proximity to FT loci enabled *cis*-regulatory changes; epigenetic silencing implicated	Photoperiod stratification (long/intermediate/short-day) limits introgression
*A. fistulosum*	Mixed/vegetative perennial	Tillering, overwintering, vegetative vigor	Elevated TFL1-like expression reinforces vegetative identity and suppresses flowering	Smaller expansion than garlic; milder sulfur profile	TE insertions and regulatory shifts associated with perennial growth	Partial reproductive isolation; limited introgression with onion (*A. ×proliferum*)
*A. sativum*^a^	Obligate vegetative (clonal)	Sterility, bulb/clove diversification	Expanded PEBP family (∼26 genes); FT/TFL1 regulatory rewiring linked to sterility and bulb formation	Largest expansions (alliinase, GGT, LFS); tandem and dispersed duplications	TE-driven genome expansion (>90% LTRs) and epigenetic regulation shape gene dosage and expression	Clonal propagation preserves traits but hampers introgression; restoring fertility is critical for breeding

a While the majority of domesticated *A. sativum* are clonally propagated and sterile, few reports highlight fertile *A. sativum* lines.

In contrast, *A. fistulosum* was domesticated for perenniality, overwintering capacity, and edible pseudostems rather than bulbs.^39^^,^^70^ Selection favored thickened stems, vigorous tillering, and vegetative identity, supported by upregulated TFL1-like genes, enhanced vascular development, and auxin/cytokinin-driven propagation networks.^39^^,^^60^ Recent resequencing of 135 accessions resolved two major genetic groups, with subpopulations across China, Central Asia, Japan, Europe, and the Americas.^39^ Northern and northeastern China emerge as centers of diversity, while Central Asian accessions show signatures of early divergence, suggesting possible independent domestication. Evidence of gene flow between regions indicates complex migration histories, while the introduction of accessions from southeastern China into Japan and beyond explains the global distribution of Group 2 lineages. Overall, these underscore rooted *Allium* domestication in a shared genomic framework that was progressively and differentially reshaped across species by directional, lineage-specific selection.

## Convergent and divergent paths of domestication

Although onion, garlic, and Welsh onion followed distinct domestication trajectories, they share convergent outcomes. In all three, selection redirected photosynthate toward edible structures – driven by sucrose transporters in interplay with several sucrose metabolism-related genes in early bulb development in onion^75^ and garlic through hormonal and transcriptional networks, enhancing carbohydrate allocation, and pseudostems in Welsh onion, where diversity in gibberellin sensitivity causes tillering capacities (Table 1).^76^ A second unifying feature is the rewiring of photoperiod and circadian pathways, particularly *FT/TFL1* homologs, which enabled adaptation to diverse latitudes and growing seasons (Table 1). Yet their reproductive strategies diverged; domesticated onion retained facultative sexuality, garlic became highly sterile^77^^,^^78^ and clonal, while a few retain partial fertility,^79^^,^^80^ and Welsh onion adopted a perennial, vegetatively dominant strategy (Table 1). These contrasts reflect ecological pressures and cultural preferences, from seed-based propagation to overwintering persistence.

Comparisons across species reveal distinct molecular routes to similar developmental outcomes. As mentioned above, in onion, domestication uncoupled bulbing and flowering via *cis*-regulatory variation at FT paralogs, suppressing *AcFT4* while enhancing *AcFT1* to promote bulb enlargement while maintaining sexuality.^71^ Welsh onion domestication, by contrast, emphasized vegetative persistence, with elevated TFL1-like expression stabilizing perennial growth and repressing flowering. Garlic represents the extreme, with ∼26 PEBP family members, extensive *FT/TFL1* and *LFY* rewiring, and sterility despite the retention of core flowering genes. Genome inflation through TE expansion (>90% LTR in garlic) further drove gene duplication and novel regulatory landscapes, with epigenetic modulation likely central to domestication-related traits.^66^^,^^81^ Photoperiod allelic variation additionally created strong reproductive barriers, restricting gene flow and complicating modern hybrid breeding.

Moreover, flavor evolution provides another axis of convergence and divergence. All three species expanded sulfur metabolism pathways, but garlic underwent the most extensive gene family duplication, aligning with its pungency and medicinal value. Onion and Welsh onion show more moderate specialization, with Welsh onion favoring milder flavor and stress-associated metabolites. Integrating genomics and metabolomics has clarified these patterns, with studies revealing genotype-specific phytochemical fingerprints, metabolite-gene associations, and even chromosomal hot spots (e.g., flavonoid biosynthesis on onion chromosome 5A).^67^ Metabolomic studies in garlic and onion further show that genetic variation interacts strongly with postharvest processing to shape phytochemical outcomes. Furthermore, holistic comparisons with other crops underscore that metabolic rewiring is a universal feature of domestication. Selection has reshaped lipid, amino acid, alkaloid, terpenoid, and anthocyanin pathways in wheat, maize, tomato, and grape, often via structural variants such as transposable element insertions or via pleiotropic regulators such as Teosinte Branched 1 (TB1). Similarly, in *Allium*, conserved upregulation of carbohydrate metabolism, CSO biosynthesis, and stress-response pathways contrasts with the lineage-specific regulation of hormones and flowering.

Taken together, the integrated genomic and metabolic evidence suggests that *Allium* domestication proceeds through convergent selection on developmental and metabolic trade-offs, while simultaneously promoting diversification at structural, regulatory, and ecological levels. This collective rewiring of conserved pathways generated the distinct domestication syndromes of garlic, onion, and Welsh onion, revealing a common evolutionary template that can inform more mechanistic, forward-looking breeding efforts.

## Future perspectives for breeding and crop innovation

A detailed understanding of genome evolution and domestication processes in *Allium* crops would provide a robust framework for crop improvement. The emerging recognition that crop metabolomes were shaped both wittingly and unwittingly has prompted a paradigm shift in crop improvement strategies. Marker-assisted selection could target genomic loci associated with bulb size, flavor, flowering time, and abiotic stress tolerance. In garlic, progress in somatic embryogenesis and haploid technology may even pave the way for restoring sexual reproduction in select genotypes, enabling hybrid development. Moreover, conserved regulatory modules – such as *FT* gene networks and sulfur metabolism clusters – offer strategic targets for genomic selection and trait introgression across *Allium* species.

The major advances and insights on crop domestication are based on genomics and transcriptomics data. This holds true especially for cereals and legumes. Genomic studies have revealed the introduction of common bean from the Andean origin to Europe, with further evidence of the adaptive integration of Andean genomic segments to the Mesoamerican-derived European genotypes.^82^ This was further supported by identifying selective signatures, namely gene losses (partial and complete), that define key adaptive genetic changes.^83^ In rice, genomics and transcriptomics have revolutionized our current understanding of rice domestication by enabling high-resolution mapping of genetic variation and gene function across diverse Oryza species.^84^ Comparative genomic analyses have identified key domestication genes, such as *Sh4* and *OsSh1*, in Asian rice (*Oryza sativa*) and their orthologs in African rice (*Oryza glaberrima*), revealing that parallel loss-of-function mutations independently contributed to the non-shattering trait essential for domestication.^85^^,^^86^ These findings highlight convergent evolutionary paths in the domestication of rice on different continents. The advancement of pan-genomic resources has further revealed extensive genetic diversity in wild relatives, such as *O. rufipogon* and *O. barthii*, which are valuable for improving stress resistance, yield, and adaptability in cultivated varieties.^87^ Additionally, precise genome editing technologies, notably CRISPR-Cas9, have enabled the neodomestication of wild species such as *Oryza alta*, allowing targeted manipulation of domestication traits including plant height, flowering time, grain size, and seed shattering, thus accelerating the development of resilient crops adapted to marginal environments.^88^ In the case of *Allium cepa,* recent advances in ballistic delivery of CRISPR/Cas9^89^ enable promising alternative venues to stable transformation^90^^,^^91^ that might facilitate gene-editing as well as potential neodomestications. Besides grain crop domestication, the domestication of crops in the Solanaceae family, particularly tomato (*Solanum lycopersicum*) and potato (*Solanum tuberosum*), has been dramatically elucidated by recent advances in genome sequencing and graph-based pangenomics. In tomato, structural variants (SVs), often overlooked in traditional genome-wide association studies (GWASs), were shown to account for a significant portion of missing heritability in complex traits such as fruit metabolites and gene expression levels.^92^ A tomato graph pangenome allowed for a 24% increase in estimated trait heritability compared to a linear reference genome, primarily due to the improved detection of SVs.^92^ One exemplary gene affected by domestication-associated SVs is *Solyc03G002957*, where a 2,628-bp deletion proximal to the gene was found to contribute significantly to gene expression heritability, an effect not captured by SNP-based models alone.^92^ These findings emphasize the role of SVs in gene regulation and phenotypic variation, critical for crop improvement.

In parallel, potato domestication and evolution have been explored through a pan-genome analysis of 44 diploid accessions, encompassing wild and cultivated varieties. The analysis revealed high-confidence SVs, many of which affect gene expression and trait variation.^93^ A striking example is the identification of a 5.8 Mb inversion near the *Soltu.DM.03G018410* gene, which encodes a beta carotene hydroxylase involved in tuber flesh color. This inversion co-segregates with the Y locus for yellow tuber flesh and may lead to linkage drag in breeding.^93^ Further, a novel domestication gene, *IT1* (*Soltu.DM.06G025210*), was shown to be essential for tuber identity in potato. Knockout of *IT1* transformed stolons into branches, highlighting its critical role in tuber development. This gene interacts with the mobile signal *SP6A*, forming a regulatory complex necessary for tuberization, an adaptation absent in tomato and non-tuberous relatives.^93^ These findings illustrate how genome structure and gene regulatory variation have driven domestication in the Solanaceae.

However, despite the fact that less economically important species, such as cauliflower^94^ have a better defined domestication, we believe that sequencing and gene editing technologies continue to advance, that the immense genetic diversity of the *Allium* genus offers unparalleled potential for a detailed refining of *Allium* domestications and ultimately serving to sustainable agriculture, climate resilience, and global food security. To this end, *de novo* domestication, leveraging genome editing technologies such as CRISPR/Cas9, now enables the targeted introduction of favorable metabolic and developmental traits into wild or semi-domesticated species,^95^^,^^96^ with reduced linkage drag and enhanced nutritional outcomes. Simultaneously, redomestication of underutilized, resilient species, particularly from the African and American centers of diversity, provides a sustainable route to diversify food systems under climate stress^97^^,^^98^ as well as wild *Allium* relatives with advantageous traits such as disease resistance or enhanced nutritional profiles.

## Acknowledgments

We thank the reviewers for their valuable suggestions and input during the reviewing process.

## Author contributions

Conceptualization, M.B.; writing – original draft, M.B.; writing – review and editing, E.A.H., A.K., A.R.F., and M.B.; supervision, M.B.

## Declaration of interests

The authors declare no competing interests.

## Declaration of generative AI and AI-assisted technologies in the writing process

During the preparation of this work, the author used ChatGPT to improve the readability and language of the article. After using these tools, the authors reviewed and edited the content as needed and take full responsibility for the content of the published article.
